# Businesses marketing purported stem cell treatments and exosome therapies for COVID-19: An analysis of direct-to-consumer online advertising claims

**DOI:** 10.1016/j.stemcr.2023.09.015

**Published:** 2023-10-26

**Authors:** Leigh Turner, Juan Ramon Martinez, Shemms Najjar, Thevin Rajapaksha Arachchilage, Jia Chieng Wang

**Affiliations:** 1 Program in Public Health, University of California, Irvine, 653 E. Peltason Drive, 2nd Floor, Irvine, CA 92697-3957, USA; 2 Sue and Bill Gross Stem Cell Research Center, University of California, Irvine, Irvine, CA, USA; 3 Department of Family Medicine, University of California, Irvine, Irvine, CA, USA

## Abstract

We identified 38 businesses advertising purported stem cell interventions and exosome products for COVID-19. These companies operated or facilitated access to 60 clinics. More than 75% of these clinics were based in the United States and Mexico. Thirty-six of the businesses marketed their stem cell and exosome products as treatments for Long COVID, six advertised them as “immune boosters,” five claimed to treat patients in the acute infection phase, and two claimed their products were preventive. The least expensive product cost $2,950, the most expensive was $25,000, and the average listed cost for patients was $11,322. The promotion of these products is concerning because they have not been approved by national regulators and do not appear to be supported by convincing safety and efficacy data.

## Associated podcast

For an associated discussion of this work, listen to the latest episode of The Stem Cell Report podcast at https://www.isscr.org/podcast/s3-e3, brought to you by the ISSCR.

## Teaser

Thirty-eight businesses operating or providing access to 60 clinics were found marketing unproven and unapproved stem cell or exosome products for COVID-19. Most of these businesses targeted patients with Long COVID; however, some companies advertised such products as preventive interventions, immune boosters, or treatments for acute infection. Regulators need to investigate businesses using misleading advertising to sell such products and stop them from engaging in such problematic commercial activity.

## Main text

During the COVID-19 pandemic, numerous unproven and unapproved medical interventions have been marketed as treatments, cures, or preventive measures for COVID-19 (Freckelton, 2020; Liu et al., 2020). Businesses selling colloidal silver, vitamin-based “immune boosters,” echinacea, elderberry elixir, ozone and hyperbaric oxygen therapy, essential oils, cannabidiol products, herbal teas, Thymosin Alpha-1, and other purported therapies have used online direct-to-consumer advertising tools to attract customers (Bramstedt, 2021; Rachul et al., 2020; Tran et al., 2021). Companies selling putative stem cell treatments and exosome therapies are among the entities promoting products that have not been approved for use or authorized for emergency use by the US Food and Drug Administration and other national regulators.

Within the global marketplace of businesses engaged in direct-to-consumer advertising of stem cell interventions and exosome products for wide range of diseases and injuries, some companies have tailored their sales pitches to the pandemic and now market these interventions as treatments for COVID-19 (Turner, 2020). Their clients have included individuals hoping to avoid infection by the SARS-CoV-2 virus, patients seeking treatment during the acute phase of COVID-19 infection, and individuals seeking relief from symptoms classified using such diagnostic categories as “Long COVID,” “Post-Acute COVID-19 Syndrome” (PACS), and “Post-COVID” (Del Rio et al., 2020; Groff et al., 2021; Nalbandian et al., 2021). Such claims have prompted concerns about patient safety, deceptive advertising representations, exploitation of vulnerable persons, and misleading appropriation of credible scientific research (Turner et al., 2021). This study examines this troubling marketplace by analyzing online representations made by businesses advertising stem cell-based interventions and exosome products as purported treatments or preventive measures for COVID-19.

Three complementary approaches were used to find businesses advertising purported stem cell interventions and exosome products for COVID-19.

First, a 2021 database capturing online representations made by 1,480 US businesses engaged in direct-to-consumer marketing of putative stem cell treatments and exosome therapies for various indications was reviewed to determine whether any of the listed companies promoted such products to treat or prevent COVID-19 (Turner, 2021). Websites of all companies listed in the database were searched for claims promoting stem cell-based interventions or exosome products as treatments or preventive measures for COVID-19. This search, conducted from November 2021 to January 2022, identified 5 US-based companies making such claims.

Second, to search for comparable businesses operating clinics located outside the United States and to investigate whether additional US companies could be identified, a series of stem cell and COVID-19-related search terms was entered into the Google search engine. Examples of search phrases include “stem cell treatment for COVID-19,” “stem cell clinic treating COVID-19,” and “exosome therapy for COVID-19.” Twenty pages of returned links were reviewed per search term, except when fewer pages were returned by the search engine. Using this search strategy, 32 newly identified businesses were found engaged in online direct-to-consumer marketing of purported stem cell or exosome treatments or preventive interventions for COVID-19.

Third, an article published relatively early in the pandemic examined ethical, regulatory, and scientific concerns associated with businesses selling unlicensed and unproven stem cell interventions for COVID-19. A supplement to the article provided links to websites of specific businesses that in Spring (2020) were selling such products directly to consumers (Turner, 2020). Websites of these businesses were reviewed to determine whether any of them remained engaged in marketing to patients stem cell-based interventions or exosome products for COVID-19. When these websites were analyzed in September 2022, one company identified in the supplement was still selling purported stem cell-based interventions for COVID-19.

Websites of identified businesses were analyzed for information concerning clinic locations, number of clinics per business, whether companies described themselves as operating their own clinics or facilitating travel to clinics run by other businesses, types of stem cell and exosome products marketed as COVID-19 therapies or preventive interventions, types of claims made about treating or preventing COVID-19, specified method or methods for delivering such products to patients, and advertised prices. This information was assembled in a dataset capturing selected online representations made by the identified businesses.

Supplementing the primary focus on businesses’ websites, social media feeds of companies using such platforms were examined to see whether they contained marketing representations connecting advertised stem cell and exosome products to the prevention or treatment of COVID-19. Key social media statements describing sale of stem cell or exosome products for treatment or prevention of COVID-19 were captured and added to the database.

Data mining, content analysis, and coding of business websites first occurred after companies were found advertising stem cell and exosome products for COVID-19. All company websites were then revisited and reanalyzed in September 2022, ensuring the data mining and content analysis process captured recent marketing representations. The research team then conducted a final fact-check in October 2022, ensuring representations made on company websites had been accurately captured, analyzed, and recorded.

**Figure 1:**
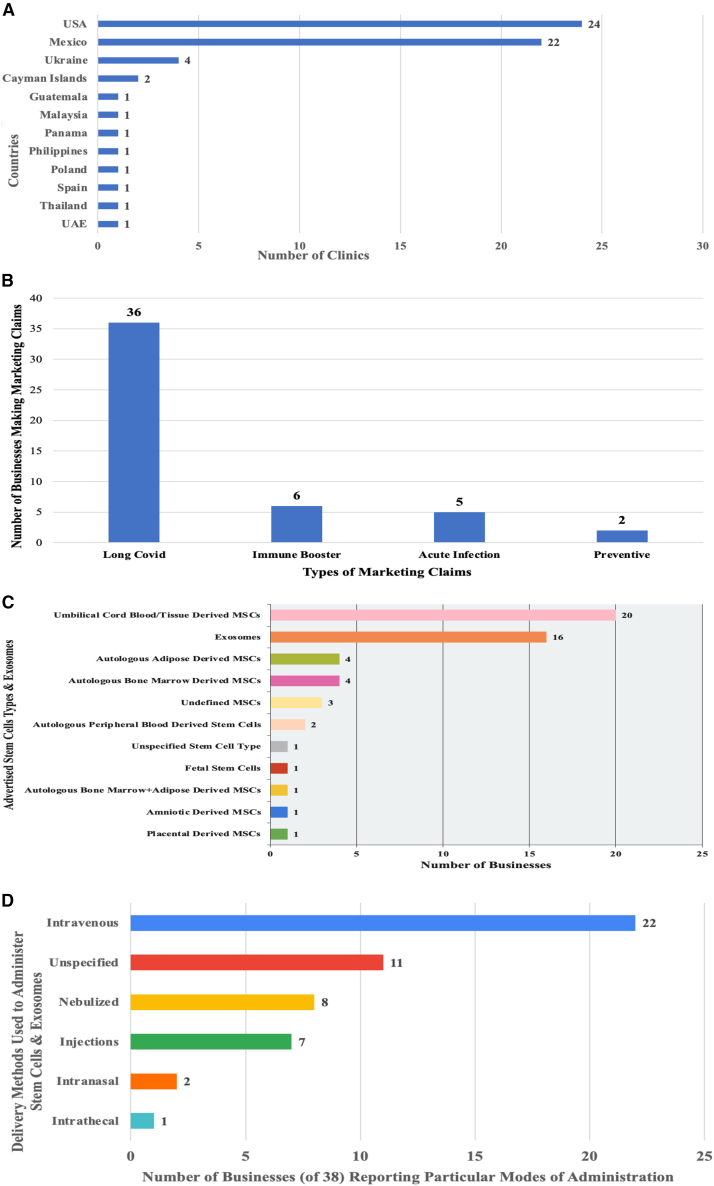
Businesses marketing stem cells and exosomes as treatments for COVID-19 (A) Geographic distribution of 60 clinics marketing stem cell-based interventions or exosome products for COVID-19. UAE, United Arab Emerites. (B) Marketing claims made by businesses selling stem cell-based interventions and/or exosome products for COVID-19 (38 total businesses). (C) Number of businesses marketing stem cell types and exosomes (of 38 businesses). MSC, mesenchymal stem cell. (D) Delivery methods used by businesses to administer stem cell-based interventions and exosome products.

Thirty-eight businesses operating or facilitating access to 60 clinics were found selling purported stem cell-based treatments or exosome therapies for COVID-19. Thirty-six businesses advertised stem cell interventions or exosome products administered at their own clinics. Two businesses were medical travel facilitators that marketed stem cell or exosome products for COVID-19 and coordinated travel to affiliate clinics. Twenty-four of the 60 clinics (40%) listed on websites of the identified businesses were in the United States, 22 (37%) were in Mexico, four (7%) were in Ukraine, and two (3%) were in the Cayman Islands. Guatemala, Malaysia, Panama, Philippines, Poland, Spain, Thailand, and the United Arab Emirates had one clinic each (1.7% per country), with the clinics collectively representing a combined market share of 13% (Figure 1A).

Thirty-six businesses (95%) marketed stem cell-based interventions or exosomes as treatments for individuals suffering Long COVID symptoms, six (16%) sold such products as “immune boosters,” five (13%) targeted individuals seeking treatment during the acute infection phase, and two (5%) claimed their products were preventive (Figure 1B). Five businesses made two distinct claims concerning their products’ ability to treat the acute phase of COVID or Long COVID, boost the immune system, or prevent transmission of SARS-CoV-2. Three businesses made three such claims.

The businesses advertised a variety of purported stem cell-based interventions and exosome products (Figure 1C). Twenty businesses (53%) sold umbilical cord blood or umbilical cord tissue-derived mesenchymal stem cells, 16 (42%) marketed exosomes, four (11%) advertised autologous adipose-derived mesenchymal stem cells, four (11%) promoted autologous bone marrow-derived mesenchymal stem cells, three (8%) advertised undefined mesenchymal stem cells, and two (5%) marketed autologous peripheral blood-derived stem cells. Amniotic-derived mesenchymal stem cells, placental-derived mesenchymal stem cells, fetal stem cells, and autologous bone marrow-derived mesenchymal stem cells combined with autologous adipose-derived mesenchymal stem cells were each advertised by one business (3% per company). One business (3%) marketed stem cells without identifying a specific cell type or source. Numerous businesses promoted more than one type of stem cell or exosome product. Twelve businesses advertised two types of stem cell or exosome products, and two businesses advertised three different types of stem cell or exosome products.

Businesses made numerous representations concerning how patients were administered stem cell-based interventions or exosome products (Figure 1D). Twenty-two businesses (58%) reported using intravenous infusions, eight (21%) claimed they nebulized their products, seven (18%) stated they provided injections, two (5%) mentioned intranasal administration without referring to nebulization, and one (3%) marketed intrathecal administration of stem cells. Eleven businesses (29%) did not specify how they administered their products. There were more modes of administration recorded than there were businesses because some businesses listed two or more methods of delivering stem cell-based interventions or exosome products. Six businesses marketed two modes of delivering such products, two businesses advertised three distinct delivery methods, and one company promoted four methods of administration.

Nine businesses provided information concerning how much they charged for the stem cell or exosome products they marketed for COVID-19. The least expensive product advertised for COVID-19 was $2,950, the most expensive was $25,000, and the average listed cost for patients was $11,322. Some prices were listed as minimum charges, suggesting that actual costs to patients might exceed base rates. Two businesses posted price ranges rather than specific prices. Where price brackets were provided, the lowest and highest rates were combined to create an average price that could then be combined with other listed prices to create an overall average price for the businesses publicizing the rates they charged.

Some businesses used social media platforms to reach prospective clients. Seven companies used Facebook to promote stem cell-based interventions or exosome products for COVID-19; four advertised them using YouTube videos. One business that used Facebook and YouTube as marketing platforms also used Twitter, Instagram, and TikTok to reach potential customers.

Thirty-eight businesses operating or facilitating access to 60 clinics were found selling stem cell-based interventions or exosome products as treatments or preventive interventions for COVID-19. These companies use marketing representations that capitalize on the potential for stem cell-based interventions and exosome products with anti-inflammatory, immunomodulatory, or regenerative properties to emerge as evidence-based therapies for patients with COVID-19. At present, however, such products remain investigational and require further evaluation in well-designed, sufficiently powered controlled clinical trials that comply with contemporary ethical, scientific, and regulatory standards (Khoury et al., 2020; 2021). Premature commercialization of stem cell-based interventions is a widespread and long-standing problem (Sipp et al., 2017). It is concerning that some businesses have tailored their direct-to-consumer marketing pitches to the COVID-19 pandemic and are selling stem cell and exosome products that are not supported by substantial evidence of safety and efficacy.

Patients suffering from Long COVID are the primary marketing target of businesses engaged in direct-to-consumer advertising of stem cells and exosome products for COVID-19. It is understandable that individuals seeking relief from shortness of breath, fatigue, “brain fog,” heart palpitations, loss of smell, and other symptoms search for interventions that might help them. Acknowledging the suffering and agency of such persons, members of this patient population are vulnerable to having their suffering, desperation, and hope exploited by entities making appealing therapeutic claims without having the scientific evidence needed to make such representations. Likewise, there is no current scientific consensus that stem cell interventions and exosome products can reliably prevent individuals from being infected by SARS-CoV-2 or “boost” their immune systems in such a manner that their risk of infection is reduced. Such marketing claims are distinct from translational research efforts that have responded to the pandemic by generating meaningful safety and efficacy data for specific stem cell interventions and exosome products.

Some national regulatory bodies have been responsive to businesses selling unapproved and unproven stem cell-based interventions or exosome products for COVID-19. For example, the US Federal Trade Commission has issued a number of warning letters to companies selling such products, and the US Food and Drug Administration has issues warning letters and untitled letters to such businesses (Turner, 2020). At least one clinician in the United States was convicted of federal charges connected to marketing stem cell products as treatments for COVID-19, and state Attorneys General Offices have also sued businesses using alleged misrepresentations to sell such products. Acknowledging these important enforcement actions, other businesses appeared to have avoided attracting the attention of regulatory bodies despite using apparently misleading claims to sell stem cell-based interventions and exosome products as putative treatments for COVID-19. This activity suggests the importance of continued vigilance by organizations such as the Food and Drug Administration, Federal Trade Commission, and other national regulators.

This study analyzes online representations made by businesses selling putative stem cell and exosome products for COVID-19; however, it likely identified only some companies operating in this space. Searches for businesses were conducted using English-language search terms and phrases. A multilingual approach might identify additional companies making comparable marketing representations using other languages. Furthermore, an existing database that listed 1,480 US businesses selling purported stem cell treatments on a direct-to-consumer basis was used as one search tool. Were comparable databases available for reviewing websites of businesses and clinics based in other countries, it is possible that more non-US-based businesses marketing stem cell and exosome products to treat or prevent COVID-19 would have been found. Proprietary algorithms powering the Google search engine might also have influenced our results. These constraints constitute study limitations.

The temporal period during which this study was conducted likely also influenced our findings. Earlier in the pandemic, before vaccines were developed, businesses marketing stem cell products for COVID-19 appear to have been more focused on making claims about treating the acute phase of infection or providing an immune boost that would reduce the likelihood of infection and the severity of symptoms (Turner, 2020). While such marketing representations persist, direct-to-consumer advertising claims now emphasize stem cells and exosomes as treatments for Long COVID. This marketing orientation seems poised to persist if individuals continue suffering long-term symptoms following infection by SARS-Cov-2 and safe and efficacious therapeutics are not developed (Davies, 2022).

Finally, we searched for businesses that had company websites and engaged in online direct-to-consumer marketing representations. Acknowledging the powerful role of the internet in helping such businesses selling their products, some companies might use other marketing platforms and not make online representations. It is conceivable that some businesses sold stem cell-based interventions or exosome products as purported treatments or preventive measures for COVID-19, and used billboards, newspaper and magazine advertisements, radio or television commercials, word-of-mouth marketing, or other techniques to promote their products. Some of these methods could be highly local in nature, and not easy to detect unless attending to a variety of marketing platforms in particular geographic regions. Our research methods were attuned to identifying online marketing representations. We therefore could have failed to identify businesses using other advertising techniques and tools to attract prospective clients.

Regulatory bodies and law enforcement agencies responsible for protecting patients and consumers from potentially harmful products and misleading marketing representations, scientific societies, patient advocacy groups, and other organizations have important roles to play in ensuring that businesses selling purported stem cells or exosome treatments or preventive interventions for COVID-19 comply with ethical, scientific, and legal standards for developing, advertising, and administering such products. Regulatory agencies in nations containing businesses engaged in noncompliant commercial and clinical activities should act to ensure compliance with existing regulatory standards. Countries with gaps and areas of interpretive uncertainty in legislation related to stem cells, exosomes, and other regenerative medicine products should develop and enforce more comprehensive regulatory structures. Further clinical research should provide insight into whether specific stem cell-based interventions or exosome products are backed by sufficient evidence of safety and efficacy related to treating or preventing COVID-19 to warrant premarketing approval or emergency use authorization by national regulators. Absent such evidence, patients are vulnerable to being exploited by businesses using direct-to-consumer online advertising to make persuasive but unjustified claims about treating or preventing COVID-19.

## Supplemental information

Data S1. Database of businesses marketing purported stem cell treatments and exosome therapies for COVID-19
